# Factors impacting caregiver burden for familial caregivers in Puerto Rico: Perspectives from 51 caregivers of people living with Alzheimer's Disease

**DOI:** 10.1002/alz70858_103595

**Published:** 2025-12-24

**Authors:** Alexandra Mendoza‐Graf, Gabriela Castro, Gilberto Ramos Valencia, Mariela Torres‐Cintrón, Ivonne Z. Jimenez Velazquez, Ilka Rios, Johan Hernandez Santiago, Beverly Weidmer, Marielena Lara, Justin W. Timbie

**Affiliations:** ^1^ RAND Corporation, Santa Monica, CA, USA; ^2^ University of Puerto Rico, School of Medicine, San Juan, PR, USA; ^3^ University of Puerto Rico, School of Medicine, San Juan, Puerto Rico, PR, USA; ^4^ RAND Corporation, Alexandria, VA, USA

## Abstract

**Background:**

Alzheimer's disease (AD) prevalence in Puerto Rico (PR) exceeds that of the mainland United States, with familial caregiving being prevalent due to strong Latino cultural traditions. Caregiving burdens significantly affect caregiver health, and Puerto Rican caregivers are among the most likely in the U.S. to report fair or poor health. This study explores the caregiving experiences of familial caregivers of AD patients in PR, identifies factors contributing to caregiver burden, and suggests improvements in policy and care delivery.

**Method:**

We conducted 51 in‐person interviews with AD patients and their familial caregivers in PR. Interviews were recorded, transcribed verbatim, and analyzed using a directed content approach. Interrater reliability was ensured using Cohen's Kappa.

**Result:**

Caregivers reported significant burdens, including constant caregiving demands and inadequate respite, exacerbated by AD progression. Key contributing factors included a lack of adult day centers and high out‐migration, limiting family support. While some municipalities offered homemaker services or part‐time nurses, availability was inconsistent. Caregivers faced challenges in accessing healthcare for patients, with difficulties in securing timely appointments and coordinating care, which often required them to manage medication lists and lab reports across providers. Additionally, some caregivers experienced challenges in navigating patients’ care when patients felt they could continue to independently attend healthcare visits, leaving caregivers unaware about certain care needs. Financial burdens related to medical and daily living costs, along with insufficient preparedness for caregiving roles, were prevalent. Further, unmet mental health support needs for caregivers were frequently expressed.

**Conclusion:**

The findings reveal the extreme burden on familial caregivers of AD patients in PR, highlighting the need for enhanced support from social networks and healthcare providers. Policies should focus on integrating caregiver wellbeing into patient care, enhancing respite support, improving healthcare access and coordination, and providing training programs for practical caregiving skills and emotional support. A family‐centered care approach that addresses both patient and caregiver needs is essential for improving the overall well‐being of those involved in AD care.

